# Contributing factors to iron deficiency anemia in women in Jordan: A single-center cross-sectional study

**DOI:** 10.1371/journal.pone.0205868

**Published:** 2018-11-05

**Authors:** Muhammad Awidi, Hisham Bawaneh, Hadil Zureigat, Muna AlHusban, Abdalla Awidi

**Affiliations:** 1 Cell Therapy Center, University of Jordan, Amman, Jordan; 2 University of Jordan Hospital, University of Jordan, Amman, Jordan; 3 Faculty of Medicine, University of Jordan, Amman, Jordan; Mahidol Oxford Clinical Research Unitl (MORU), THAILAND

## Abstract

**Objectives:**

This study aimed to understand the impact of iron deficiency anemia in female users of a hematology service in a developing country.

**Design:**

Retrospective cross-sectional study of adult and adolescent women with iron deficiency anemia who presented to a hospital department of hematology.

**Setting:**

A tertiary university hospital inpatient and outpatient hematology service.

**Participants:**

All female patients who were ≥13 years of age with confirmed iron deficiency anemia and received hospital hematology services.

**Results:**

A total of 208 patients were enrolled and analyzed in the registry. The mean age of the patients was 41.4 years (range, 14–82). A total of 195 patients had anemia that was moderate or severe according to the World Health Organization anemia classification with 13 patients having mild anemia. A total of 108 patients had comorbidities, which were primarily endocrine and cardiovascular. Iron deficiency anemia was associated with very heavy (n = 56, 30%) or heavy menses (n = 84, 45%) in 140 patients and was associated with poor (<200 g/week of red meat) (n = 101, 54%) or very poor (vegan, strict vegetarian) nutrition (n = 34, 18%) in 135 patients. A total of 101 patients had a previous pregnancy history with a mean of six previous pregnancies (range, 1–11 pregnancy episodes per patient). Blood film was performed on all patients; only four had a picture consistent with thalassemia minor.

**Conclusion:**

Iron deficiency anemia is caused by multiple factors. Heavy menses and low consumption of red meat were found to be associated with the severity of anemia. Our findings may be useful for healthcare planners and policy makers in increasing efforts to reduce the prevalence and severity of iron deficiency anemia among women in Jordan.

## Introduction

Anemia is one of the most common health conditions worldwide, with a reported global prevalence of 32.9% in 2010 [1]. Globally, half of all anemia cases are due to iron deficiency anemia (IDA) [2, 3] and IDA remains the leading cause and second leading cause of anemia in women and men, respectively [1, 4]. IDA is reported to contribute to more than 800,000 deaths per year worldwide, in addition to causing physical and mental disability in children and older adults. Furthermore, IDA contributes to reduced productivity in adults [2, 3]. The health burden of IDA is an enormous cause of year life lived with disability (YLD) and is still a major global health concern [1]. According to the World Health Organization (WHO) 1993–2005 report on anemia, the prevalence of IDA in non-pregnant women in the eastern Mediterranean region is 32.4% (range, 29.2–35.6) [5]. A national household-level micronutrient survey was conducted in Jordan in 2010 and 2002 and collected the data of 2,026 non-pregnant women aged 15–49 years; the prevalence of iron deficiency and IDA were found to be 35.2% and 19.6%, respectively [6].

Etiologies of IDA vary among different populations, depending on factors such as age, gender, socioeconomic status, and ethnicity. In premenopausal women, studies show that excessive menstruation is the most common etiology of IDA [7]. On the other hand, occult bleeding from the gastrointestinal (GI) tract is the main cause of IDA in men and postmenopausal women [8].

Jordan is a low-middle income country [9] and based on statistics from other countries and the abovementioned reports, the financial burden is assumed to be quite significant. However, Jordan currently lacks formal educational and health policies aimed at addressing the issue of IDA. Therefore, the aim of this study was to understand the impact of iron deficiency anemia in female users of a hematology service in Jordan using data from a registry of women with IDA.

## Methods

This study was conducted according to the guidelines laid down in the Declaration of Helsinki and all procedures involving human subjects/patients were approved by the University of Jordan Hospital Institutional Review Board. Written informed consent was obtained from all study participants or the legal guardian of patients younger than the age of 18.

All patients admitted or referred to the adult hematology service of Jordan University with a diagnosis of IDA were enrolled in a hospital-based registry. The hospital policy assigns patients aged ≥13 years to the adult hematology services. A total of 208 patients with IDA were enrolled during the registry's first year, from April 1, 2016 to March 31, 2017.

### Participant recruitment and data collection

Non-pregnant, female patients older than 13 years of age were included for analysis in this study. To be diagnosed with IDA, the patient must have a hemoglobin level of <12 g/dL (if non-pregnant) with either ferritin <30 μg/l, and/or two of the following parameters: mean corpuscular volume <80 fL and red cell distribution width (RDW/CV) >14.5%, or blood film appearance typical of iron deficiency anemia (e.g. microcytosis, hypochromia, anisocytosis, poikilocytosis) [10].

The following data was captured by the registry: 1) general patient profile: name, national identification number, gender, age, smoking status and co-morbid conditions; 2) laboratory tests: complete blood count (i.e. hemoglobin level, white cell count [WBC] and WBC differential, mean corpuscular volume, red cell distribution width, platelet count), reticulocytes count, ferritin, serum iron, total iron binding capacity, serum B_12_, folate level, lactate dehydrogenase, direct and indirect anti-globulin tests (Coombs test when needed), hepcidin level (when available), soluble transferrin receptor (sTf) (when available), blood film, hemoglobin electrophoresis (when applicable), celiac disease tests (when applicable; tissue transglutaminase antibodies A and G [tTG-IgA, tTG-IgG] were considered positive if titer was >10.0 U/mL and or >9.0 U/mL, respectively) and fecal occult blood; 3) gastrointestinal endoscopy: upper gastrointestinal endoscopy (if performed) with a biopsy from the abnormal areas and colonoscopy (if performed) with a biopsy from the abnormal areas; 4) documentation of menstrual periods: assessment of menstrual blood loss was done using a pictorial chart based on patient’s history and was considered regular if menstrual flow occurred every 21–35 days and lasted two to seven days with pads lightly soiled [11] heavy if there were blood clots or bleeding lasting more than seven days with pads moderately soiled, and very heavy if multiple pads were saturated during the menstrual flow period each day or if the patient had a gynecological procedure such as myomectomy or dilation and curettage to deal with menorrhagia; 5) assessment of nutritional status: iron nutritional status was assessed based on the average consumption of red meat or processed red meat per week, and was divided into three main categories: a) poor iron nutritional status (strict vegetarian or vegan patients with no red meat consumption), b) poor iron nutritional status (patients consuming red meat or processed red meat [less than 200 g per week] once a week or less), c) normal iron nutritional status (patients who consume red meat or processed red meat [350 g per week] twice per week or more [12, 13]; 6) treatment: if given, blood transfusion units were documented and intravenous iron treatment was also documented; and 7) the number of pregnancies was documented regardless of pregnancy outcome (a gravida). The number of pregnancies reaching viable gestational age was also recorded (parity). Gravida was considered positive if pregnancy was confirmed by serum human chorionic gonadotropin (hCG) and/or ultrasonography testing. Gravida was categorized as none (never pregnant), multigravida (less than 5 pregnancies) or grand multigravida (≥5 pregnancies). Parity was defined as fetus reaching a gestational age ≥20 weeks [13]. Parity was also categorized as none (never pregnant or fetus not reaching the gestational age of ≥20 weeks), multiparous or grand multiparous (<5 or ≥5 fetuses reaching the age of viability, respectively) [14].

### Iron treatment

During the one-year study period, three preparations of parenteral iron were available in the hospital: ferrous sucrose, iron dextran complex, and ferric carboxymaltose. Oral iron treatment available at the hospital during the study period was either ferrous gluconate or ferrous sulfate. During the median follow up period of eight weeks, data gathered included clarification of the cause of IDA, changes in blood parameters, and ferritin level. Oral iron or multivitamins containing iron at the time of presentation to the hematology clinic was not captured.

### Statistical analysis

Data were coded, entered into a computer, and analyzed using STATA statistical software (StataCorp LLC, College Station, TX, USA). Data are expressed as mean and range unless otherwise stated. Frequency distributions, as well as one- and two-way tabulations, were obtained. Chi-square analysis was performed to test for differences in proportions of categorical variables between two or more groups. In 2X2 tables, the Fisher's exact test (two-tailed) replaced the chi-square test if the assumptions underlying chi-square were violated, namely in the case of small sample size or where the expected frequency was less than five in any of the cells. Statistical significance was determined by a *P*-value < 0.05.

### Patient and public involvement

Patients were not involved in the recruitment or analysis of this study.

## Results

### Cohort characteristics

The registry gathered information on 223 patients who either presented to the hematology outpatient clinic or were admitted to the hospital for IDA. Of those, 15 male patients were excluded, and the remaining 208 female patients fit the inclusion criteria and were included in this study (Table 1).

**Table 1:** Patient characteristics at presentation.

Patients Attributes	Results
Mean age, ±SD (range), years	41∙4 ± 17∙3(14–82)
Mean Hemoglobin concentration ±SD (range), g/dL	8∙54 ± 1∙5(4∙4–11∙9)
Previously pregnant (n = 101)	
Mean Gravida ±SD, (range), (n = 101)	6 ±2∙5, (1–11)
Mean Parity ±SD, (range), (n = 100)	5 ± 2∙2 (1–11)
No comorbidities, n (%)	100 (48∙07)
Smoking status (n = 208)	
Smokers (n = 15), %	7∙2
Non-smokers (n = 193), %	92∙8
Comorbidities^1^ (n = 108), %	
Endocrine (n = 43)	20∙67
Cardiovascular (n = 38)	18∙27
Musculoskeletal (n = 7)	3∙37
Gastrointestinal (n = 5)	2∙4
Pulmonary (n = 5)	2∙4
Others (n = 10)	4∙81
Menses^2^ (n = 188), %	
Regular (n = 48)	25∙53
Heavy (n = 84)	44∙68
Very heavy (n = 56)	29∙79
Nutrition (n = 185), %	
Very poor (n = 34)	18∙38
Poor (n = 101)	54∙59
Balanced (n = 50)	27∙03

^1^Endocrine:Hypothyroid,Diabetes Mellitus, Dyslipidemia

Cardiovascular: Hypertension, atrial fibrillation, Deep Vein Thrombosis, ischemic heart disease cardiomyopathy

Musculoskeletal: Rheumatoid arthritis, Sjogren syndrome, Systemic Lupus Erythematosus, Knee osteoarthritis, osteoporosis and Familial Mediterranean Fever

Gastrointestinal: Gastric ulcers, Hepatitis B and Ulcerative Colitis

Pulmonary: Asthma, Lung fibrosis

Others: Thalassemia minor, chronic kidney disease, epilepsy, migraine and cerebrovascular events

^2^ 20 patients were post-menopausal and didn’t report menses

All female patients were anemic with a mean Hgb of 8.54 g/dL (range, 4.4–11.9 g/dL). The mean age of the study participants was 41.4 years (range, 14–82 years); the majority of patients were below the age of 50 years (73.08%, n = 152). Of the 208 patients, 100 (48.07%) with IDA were otherwise healthy, while 108 patients had comorbidities. Endocrinopathies comprised the majority of comorbid conditions (20.67%, n = 43), followed by cardiovascular conditions (18.27%, n = 38), musculoskeletal conditions (3.37%, n = 7), gastrointestinal conditions (2.4%, n = 5), and pulmonary conditions (2.4%, n = 5). Other conditions comprised 4.81% (n = 10). Fifteen patients were smokers (7.2%); their data was included in this analysis.

Menses was reported by 188 patients (90%), and was described as regular (25.53%, n = 48), heavy (44.68%, n = 84), or very heavy (29.79%, n = 56). Menses severity was found to be associated with the severity of anemia (*P* < 0.05). Nutritional status was assessed in 185 patients and was found to be very poor (18.38%, n = 34), poor (54.59%, n = 101), or normal (27.03%, n = 50). Of the 208 patients, 107 (51.4%) were never pregnant and 101 (48.6%) patients reported current or previous pregnancy with a mean of six pregnancies (range, 1–11; SD ± 2.6). A total of 100 pregnancies reached ≥20 weeks of gestation with a mean number of 5 pregnancies (range, 1–11; SD ± 2.2) as shown in Table 1.

### Initial assessment

All patients received liver and kidney function tests to rule out the possibility of anemia secondary to a hepatic or renal cause. Oral iron or multivitamins containing iron at the time of presentation to the hematology clinic was not captured. Of 208 patients who initially presented to the hospital, 108 (51.92%) were treated as outpatients and 100 (48.08%) were admitted to the hospital. These patients presented with either mild (6.25%, n = 13), moderate (55.29%, n = 115), or severe anemia (38.46%, n = 80) based on the WHO anemia classification of severity [1]. One patient, diagnosed with colon adenocarcinoma, had a platelet count and serum ferritin of 633.10^3^/μL and 635 ng/mL respectively. Serum iron, ferritin, and total iron binding capacity were often abnormal among all patients (Table 2).

**Table 2:** Laboratory evaluation at initial presentation, anemia severity defined based on hemoglobin (Hb) concentration using WHO anemia classification (*mild*:11–11.9, *moderate*:8–10.9, *severe*: less than 8).

Laboratory parameter	Value at initial presentation*
Hemoglobin concentration, g/dL (n = 208)	8∙47 (7.38–9.70)
Anemia Severity (n = 208), %	
Mild (n = 13)	6∙25%
Moderate (n = 115)	55∙29%
Severe (n = 80)	38∙46%
Mean corpuscular volume, fL (n = 208)	66.4 (62.50–72.15)
Red cell distribution width, CV % (n = 208)	18.95 (17.3–20.8)
Platelet count, 10^3^/μL (n = 208)	333.5 (268.5–404)
Serum ferritin, ng/mL (n = 208)	6.67 (4.01–13.30)
Iron, μg/dL (n = 172)	20.45 (16.32–26.96)
Total iron binding capacity, μg/dL (n = 163)	392 (358–431)
Serum B12, pg/mL, (n = 183)	298 (229–414)
Serum Folate, ng/mL, (n = 156)	7.30 (5.00–10.65)
Lactate dehydrogenase, U/L, (n = 136)	346 (310–414.50)
Fecal occult blood test (n = 60), %	
Positive (n = 10)	16∙66%
Negative (n = 50)	83∙33%
Anti-transglutaminase antibodies (ATA), (n = 41), %	
Positive (n = 9)	21∙95%
Negative (n = 32)	78∙05%
Tissue Transglutaminase (tTG) Antibodies, IgA, (n = 75), %	
Positive (n = 9)	12∙00%
Negative (n = 66)	88∙00%
Tissue Transglutaminase (tTG) Antibodies, IgG, (n = 62), %	
Positive (n = 5)	8∙06%
Negative (n = 57)	91∙94%

* Median (Interquartile range)

Data of serum hepcidin and serum transferrin receptor were not available during the first year of the registry. Blood film tests were performed for all patients; only four patients had results consistent with thalassemia minor. Total iron binding capacity was measured in 163 patients, with a mean of 391.9 μg/dL (range, 173–566). Vitamin B_12_ and folate tests were performed when indicated, with a mean of 376.6 ng/L (range, 92–2000; n = 183) and 8.55 nmol/L (range, 1.7–85.4; n = 156), respectively. Lactate dehydrogenase levels were assessed in 136 patients and had a mean of 371.45 U/L (range, 214–818). Direct Coombs testing was positive in 2 of 77 patients (2.6%). Fecal occult blood testing was performed in 60 patients and was found to be positive in 10 patients (16.6%) and negative in 50 patients (83.4%). A total of 61 patients had upper GI complaints and underwent an upper GI endoscopy (29.33%, n = 61); of those, 48 (78.69%) had a positive pathological finding (gastric ulcers, gastritis or *H*. *pylori* infections). Colonoscopy was indicated in 42 patients (20.19%), while colon pathology was found in 23 patients (54.76%; hemorrhoids, polyps, or malignancy). Celiac tTG testing was performed in 41 patients, with positive results in nine patients (22%). Although 75 patients were tested for celiac IgA, results were positive in only nine patients. Lastly, celiac IgG was measured in 62 patients and was positive in only five patients (Table 2).

Of the 208 patients, the cause of anemia was identified in 200 patients by history, physical examination, and laboratory testing. The most common isolated cause of anemia was menses (18.27%, n = 38) followed by nutrition (11.54%, n = 24). Gastrointestinal etiology accounted for a minority of cases (8.65%, n = 18) and adenocarcinoma of the colon was the

source of anemia in a small subset of patients (1.44%, n = 3). More than half of the cases of anemia in this study was due to a combination of the above causes (56.25%, n = 117) (Table 3).

**Table 3:** Etiology and treatment of anemia, Intravenous (IV) formulation was given as ferrous sucrose or iron dextran complex or ferric carboxymaltose, Oral treatment was either ferrous gluconate or ferrous sulfate.

	Hemoglobin concentration categories ^1^ [n, (%)]			P value
	Mild	Moderate	Severe	
Etiology of anemia (n = 200)				
Menses (n = 38)	3, (7.89)	23, (60.53)	12, (31.58)	0.61
Nutrition (n = 24)	1, (4.17)	15, (62.5)	8, (33.33)	0.73
Gastrointestinal (n = 18)	3, (16.67)	8, (44.44)	7, (38.89)	0.15
Malignancy (n = 3)	0, (0)	1, (33.33)	2, (6.67)	0.58
Combined (n = 117)	6, (5.13)	66, (56.41)	45, (38.46)	0.74
Menses Severity (n = 188)	
Regular (n = 48)	2, (4.17)	22, (45.83)	24, (50)	0.027
Heavy (n = 84)	7, (8.33)	43, (51.19)	34, (40.48)	
Very heavy (n = 56)	2, (3.57)	41, (73.21)	13, (23.21)	
Treatment (n = 208)				
IV (n = 147)	7, (4.76)	71, (48.3)	69, (46.94)	<0.01
Oral (n = 61)	6, (9.84)	44, (72.13)	11 (18.03)	
Gravida^2^ (n = 208)				
None (n = 107)	5, (4.67)	60, (56.07)	42, (39.25)	0.85
Multigravida (n = 31)	2, (6.45)	16, (51.61)	13 (41.94)	
Grand multigravida	6, (8.57)	39, (55.71)	25 (35.71)	
Parity^3^ (n = 208)				
None (n = 108)	5, (4.63)	61, (56.48)	42, (38.89)	0.56
Multiparous (n = 43)	5, (11.63)	21, (48.84)	17, (39.53)	
Grand multiparous (n = 57)	3, (5.26)	33, (57.89)	21, (36.84)	

^1^ WHO anemia classification: Severe (<8 g/dL), Moderate (10–9.8 g/dL), Mild (11.9–11 g/dL)

^2^ Multigravida defined as <5 pregnancies, Gran multigravida as ≥ 5 pregnancies

^3^ Multipara defined as <5 pregnancies after 20 weeks of gestation, Grand multiparous as ≥5 pregnancies after 20 weeks gestation

### Treatment and follow-up

Transfusion of packed red blood cells (pRBC) was performed in 59 patients (28.37%) and ranged from 1–5 units. Transfusion was initiated in all symptomatic patients. Treatment regimens were commenced based on the judgment of the treating physician, patient’s weight and the severity of anemia determined the dose used. All patients received iron, with the majority receiving parenteral iron (70.67%, n = 147) and the remainder receiving oral iron (29.33%, n = 61). Treatment modality was associated with the severity of anemia at presentation (*P* < 0.01). Of patients with mild anemia, 53.85% (n = 7) were intolerant to oral iron therapy and received parenteral iron and the remaining 46.16% (n = 6) received oral iron. Of patients with moderate anemia, 61.74% received parenteral iron (n = 71) and 38.26% (n = 44) received oral iron. Of patients with severe anemia, 86.25% (n = 69) received parenteral iron, while the remaining patients (13.75%; n = 11) received oral treatment (Table 3).

Approximately one-third of patients (n = 65) did not seek scheduled follow-up care after their initial evaluation and treatment. Hence, the subsequent clinical course of these patients is unknown. The remaining 143 patients had a median follow up of eight weeks (range, 2–16). Repeated laboratory testing revealed mean Hgb and MCV values increasing to 11.9 g/dL and 78.22 fL, respectively.

## Discussion

Few previous studies have focused on cases of IDA presenting to tertiary care hospitals. This group of patients represents the end of the IDA spectrum. In Jordan, IDA has previously been studied in the general population and in pregnant women [6, 15]. Pregnant women and children below the age of 13 years were not included in the study. Furthermore, this registry reflects the real-life practices of the Jordanian population and thus provides valuable insight into the possible causes of IDA in adult and adolescent female patients. In settings other than Jordan, IDA in hospital patients has been examined by several retrospective studies [16, 17]. Bach et al. studied the prevalence and possible causes of IDA in a retrospective cross-sectional study of all inpatients and outpatients aged ≥64 years in a large European university hospital [18] and concluded that anemia was frequently diagnosed in elderly patients. Furthermore, their findings identified nutritional deficiencies, chronic inflammation, and renal impairment as significant causes of IDA in the elderly population. Hugh et al. studied IDA in a Swiss tertiary hospital and concluded that iron deficiency is common in internal medicine with possibly as much as two-thirds of cases remaining undiagnosed. The CARMES-1 registry in Italy, of IDA in elderly patients with heart failure [17] concluded that IDA is common in heart failure, and that the required corrective measures are not always taken.

This study is the first epidemiological study of adult and adolescent hospital patients with IDA in Jordan. We intend to continue gathering data in the registry developed for this study for a total of five years; this report evaluates the findings of the first year. Our study population was comprised of women and adolescents aged ≥13 years, and the majority were within the menstruating age range. We found that heavy menstruation and red meat consumption were the leading factors associated with IDA, and accounted for 75% and 70% of the cases respectively (Table 1). This finding is consistent with a previous study in a developed country [16]. Complex comorbid conditions were observed in slightly more than half of our patients, and were primarily endocrinopathies with the majority of those being diabetes mellitus. Cardiovascular conditions were the second most commonly-observed comorbidity; of these, heart failure was the most common, a finding which is consistent with that of the CARMES-1 registry [17]. Gastrointestinal comorbidities were not commonly observed in our patients.

Interestingly, the severity of anemia in our study population was not related to repeated pregnancies or parity: moderate and severe anemia was observed equally in multigravida and grand multigravida women when compared to nulligravida women (Table 2). This, however, may be because multigravida and grand multigravida women were more likely to be under the care of obstetricians and therefore took the corrective measures needed for IDA.

IDA in nulligravida menstruating women likely reflects a lack of awareness of the importance of taking corrective measure to prevent IDA. It may also reflect that these groups of patients do not seek timely medical attention. The rate of premarital sex in nulligravida women in Jordan is low. Accordingly, the use of oral contraceptives in this population is low. This may partially account for the severity of menstrual blood loss.

The main limitation of this study is that it is focused on hospital patients in the adolescent and adult populations and is not structured to include male patients, limiting the generalizability to that population. Furthermore, data from only a small number of patients is available, as the registry was established a year ago. However, accrual of data from additional patients is ongoing, and data from a larger population will be available by the end of the five-year period. Despite these limitations, we believe that patient baseline characteristics in the coming years will be similar, given the prevalence of the disease among women in Jordan. Additionally, the hospital in which the registry is based receives patients from across the country; thus, it is likely that the national population is fairly represented.

## Conclusion

Based on our findings, IDA appears to be a significant health problem affecting adult and adolescent women in Jordan. Menstrual blood loss was most significantly associated with IDA, either as an isolated cause or as a contributing factor. New innovative policies are needed to support timely education among this population. Future epidemiological studies are needed to support the optimization of efforts to improve the health of adult and adolescent women with IDA in Jordan.

## Supporting information

S1 Dataset Contributing factors to iron deficiency anemia in women in Jordan: A single-center cross-sectional study. (XLS)
